# Correction to: Unraveling the small proteome of the plant symbiont *Sinorhizobium meliloti* by ribosome profiling and proteogenomics

**DOI:** 10.1093/femsml/uqaf004

**Published:** 2025-03-28

This is a correction to: Lydia Hadjeras *et al*., Unraveling the small proteome of the plant symbiont *Sinorhizobium meliloti* by ribosome profiling and proteogenomics, *microLife*, Volume 4, 2023, https://doi.org/10.1093/femsml/uqad012

Supplementary tables were omitted from the version originally published and have been made available online.

